# Galactose-deficient IgA1 and the corresponding IgG autoantibodies predict IgA nephropathy progression

**DOI:** 10.1371/journal.pone.0212254

**Published:** 2019-02-22

**Authors:** Dita Maixnerova, Chunyan Ling, Stacy Hall, Colin Reily, Rhubell Brown, Michaela Neprasova, Miloslav Suchanek, Eva Honsova, Tomas Zima, Jan Novak, Vladimir Tesar

**Affiliations:** 1 General Teaching Hospital, 1st Faculty of Medicine, Charles University, Department of Nephrology, Prague, Czech Republic; 2 Department of Microbiology, University of Alabama at Birmingham, Birmingham, Alabama, United States of America; 3 Longhua Hospital, Shanghai University of Traditional Medicine, Shanghai, China; 4 Department of Medicine, University of Alabama at Birmingham, Birmingham, Alabama, United States of America; 5 Jan Evangelista Purkyne University in Ústí nad Labem, Faculty of Environment, Ústí nad Labem, Czech Republic; 6 Institute of Clinical and Experimental Medicine, Department of Pathology, Prague, Czech Republic; 7 General Teaching Hospital, 1^st^ Faculty of Medicine, Charles University, Institute of Medical Biochemistry and Laboratory Diagnostics, Prague, Czech Republic; University of Mississippi Medical Center, UNITED STATES

## Abstract

**Background:**

IgA nephropathy (IgAN), the most common primary glomerulonephritis worldwide, has serious outcomes with end-stage renal disease developing in 30–50% of patients. The diagnosis requires renal biopsy. Due to its inherent risks, non-invasive approaches are needed.

**Methods:**

We evaluated 91 Czech patients with biopsy-proven IgAN who were assessed at time of diagnosis for estimated glomerular filtration rate (eGFR), proteinuria, microscopic hematuria, and hypertension, and then followed prospectively. Serum samples collected at diagnosis were analyzed for galactose-deficient IgA1 (Gd-IgA1) using new native-IgA1 and established neuraminidase-treated-IgA1 tests, Gd-IgA1-specific IgG autoantibodies, discriminant analysis and logistic regression model assessed correlations with renal function and Oxford classification (MEST score).

**Results:**

Serum levels of native (P <0.005) and neuraminidase-treated (P <0.005) Gd-IgA1 were associated with the rate of eGFR decline. A higher relative degree of galactose deficiency in native serum IgA1 predicted a faster eGFR decline and poor renal survival (P <0.005). However, Gd-IgA1 has not differentiated patients with low *vs*. high baseline eGFR. Furthermore, patients with high baseline eGFR that was maintained during follow-up were characterized by low serum levels of Gd-IgA1-specific IgG autoantibodies (P = 0.003).

**Conclusions:**

Including levels of native and neuraminidase-treated Gd-IgA1 and Gd-IgA1-specific autoantibodies at diagnosis may aid in the prognostication of disease progression in Czech patients with IgAN. Future tests will assess utility of these biomarkers in larger patients cohorts from geographically distinct areas.

## Introduction

IgA nephropathy (IgAN) is the most common primary glomerulonephritis worldwide; diagnosis requires evaluation of a renal biopsy specimen [1]. Clinical risk factors predicting poor prognosis include time-averaged proteinuria, hypertension, decreased estimated glomerular filtration rate (eGFR) [2, 3], and Haas’ score for crescents, as well as histological lesions characterized by the Oxford classification (MEST score) [1, 4, 5]. IgAN is proposed to be an autoimmune disease with a multi-hit pathophysiological process [6], influenced by genetic and environmental factors [2, 7–10]. Key role in the multi-step process is played by galactose-deficient IgA1 (Gd-IgA1), the main autoantigen recognized by IgG autoantinbodies [6]. Notably, serum levels of Gd-IgA1, assessed using a lectin assay with neuraminidase-treated IgA1, are elevated in most patients with IgAN [11] and predict disease progression in Chinese patients [12]. Testing of serum levels of Gd-IgA1 may aid in predicting disease progression as it is found elevated in IgAN patients compared to controls [13–15].

We tested a hypothesis that a combination of clinical, biochemical (serum Gd-IgA1), and histological markers improves the assessment of disease progression. We recruited 91 European Czech patients with biopsy-proven IgAN with a median follow-up 3.5±1.1 years since diagnosis. Notably, we used two assays to measure serum Gd-IgA1 levels: the current standard method that measures Gd-IgA1 after treatment with neuraminidase and a modified protocol that measured native Gd-IgA1 (without neuraminidase). Our results showed that serum levels of native and neuraminidase-treated Gd-IgA1 were associated with the rate of eGFR decline. However, Gd-IgA1 has not differentiated patients with low *vs*. high baseline eGFR. Therefore, we also assessed Gd-IgA1-specific IgG autoantibodies and have shown that patients with high baseline eGFR that was maintained during the follow up had low serum levels of these autoantibodies. Thus, early analysis of Gd-IgA1 and Gd-IgA1-specific autoantibodies may aid in the assessment of risk of disease progression.

## Materials and methods

### Subjects and samples

Serum was collected from 91 Czech patients at the time of diagnosis of IgAN, before renal biopsy was performed, and stored at -80°C. Clinical and laboratory data are summarized in **S1 and S2 Tables**. The study was approved by the Ethics Committee of the General Teaching Hospital in Prague, Czech Republic. Written informed consent was obtained.

### Assays for serum IgA, Gd-IgA1 and Gd-IgA1-specific IgG autoantibodies

IgA was measured by ELISA and the levels of Gd-IgA1 by lectin ELISA with a lectin from *Helix aspersa* (Sigma; HAA) specific for terminal GalNAc [16, 17]. The original method [11] used neuraminidase to remove sialic acid from IgA1 *O*-glycans to determine IgA1 with terminal GalNAc residues, including those that were sialylated. Here, we assessed lectin binding to neuraminidase-treated as well as to native (*i*.*e*., not neuraminidase-treated) IgA1. The latter assay measured IgA1 with terminal (*i*.*e*., not sialylated) GalNAc residues. IgA1 has core 1 *O*-glycans, *i*.*e*., GalNAc-galactose disaccharide that may contain sialic acid attached at one or the other saccharide or both sugars can be sialylated. GalNAc-attached sialic acid is α2,6-linked whereas galactose-attached sialic acid is α2,3-linked. Some O-glycan can miss galactose, *i*.*e*., have terminal GalNAc. These terminal GalNAc moieties are recognized by HAA lectin. However, sialylation of GalNAc blocks HAA binding [18]. Moreover, sialylation of galactose in GalNAc-galactose disaccharides neighboring the sites with terminal GalNAc impairs access of HAA lectin to terminal GalNAc moieties. Removal of sialic acid from Gd-IgA1 by neuraminidase treatment restores HAA reactivity. These results together suggest that binding of a GalNAc-specific lectin to Gd-IgA1 is modulated by sialylation of GalNAc as well as galactose in the clustered IgA1 *O*-glycans and that HAA assay using native IgA1 (*i*.*e*., not treated with neuraminidase) and IgA1 pre-treated with neuraminidase will provide two types of assessment of Gd-IgA1. Serum levels of galactose-deficient IgA1 were expressed in units defined as the ratio of OD determined for the individual sample and the OD for a standard Gd-IgA1 protein. 100 U of Gd-IgA1 was defined as OD of 100 ng of Gd-IgA1 standard. Levels of serum IgG autoantibodies specific for Gd-IgA1 were measured using ELISA with Gd-IgA1 as an antigen, as described [19]. Specifically, Gd-IgA1 (2.5.ug/ml) was directly coated on ELISA plates, blocked by 1% BSA-Tween, and then probed with IgG using serum diluted 500-fold. One unit was defined as IgG binding to Gd-IgA1 with OD (@490 nm) equal to 1.

### Statistical analyses

We used discrimination analysis with transformed variables (*i*.*e*., the principal) to predict a membership in a group or category based several continuous variables (*i*.*e*., values for biochemical, clinical, pathological markers) [20]. To verify the correct discriminant ability, confusion matrix was constructed, which resulted in classifying each of the objects in those categories to estimate selectivity and specificity of the combined biomarker tests. Logistic regression was used to link the occurrence or non-occurrence of an event to explanatory variables. We have used logistic regression with program XLSTAT (www.xlstat.com) to validate the results of the discriminant analysis and for the calculation of ROC curves in the case of two groups.

## Results and discussion

Sera were analyzed for IgA and Gd-IgA1, and eGFR was calculated using MDRD (Modification of Diet in Chronic Renal Disease) formula. During the follow-up period, the patients exhibited three outcomes, based on renal function at the end of follow-up and its change since diagnosis: 1) non-progressors (n = 70, with stable renal function during follow-up), 2) progressors (n = 7, defined by the decline of eGFR ≥50% from baseline, but with eGFR >15 ml/min/1.73 m^2^ at the end of follow-up), and 3) patients who reached end-stage renal disease during follow-up (n = 14, defined as those with serum creatinine >400 μmol/L at diagnosis, eGFR <15 ml/min/1.73 m^2^ at the end of follow-up, or those who started renal replacement therapy) (**S3 Table**). Discrimination analysis was performed for eGFR, serum IgA, and serum Gd-IgA1 (without or with neuraminidase). eGFR alone as well as together with IgA and Gd-IgA1 divided the cohort of 91 patients in three groups (non-progressors, progressors, and ESRD) (**S1 Fig, S3 Table**). Fourteen patients with IgAN who reached ESRD during the follow-up period had higher relative degree of galactose deficiency measured using native IgA1, *i*.*e*., without neuraminidase treatment (U/1 μg IgA) (**S4 Table, S2 Fig**) but not serum Gd-IgA1 (U/1 μg IgA) with neuraminidase treatment.

However, this approach did not differentiate patients with stable *vs*. progressive disease. Therefore, we next assessed the remaining subjects who did not reach ESRD during the follow-up (n = 77). In these subjects, progressors had higher serum levels of Gd-IgA1 in both assays (with or without neuraminidase) than non-progressors (**Table 1**).

**Table 1:** Median test (Mood test) for non-progressor [n = 70] *vs*. progressor [n = 7] groups.

Variable	p value***
**S creat**	**0.045**
**eGFR**	**0.049**
**IgA (μg/mL)**	0.155
**Gd-IgA1 (U/1** μg IgA)*	0.155
Gd-IgA1 (U/mL)*	**0.005**
**Gd-IgA1 (U/1** μ**g IgA)** **	0.155
Gd-IgA1 (U/mL)**	**0.005**

* serum Gd-IgA1 without neuraminidase pretreatment

** serum Gd-IgA1 with neuraminidase pretreatment

***p values means the risk to reject the null hypothesis (the medians are all equal). Confidence level is 90% (cut-off 0.1). Bold numbers indicate statistically significant P values.

S-creat, serum creatinine (μmol/L); eGFR (MDRD, mL/min/1.73 m^2^)

Next, we assessed potential correlations of histological findings (Oxford MEST classification of renal biopsy specimens) with clinical and biochemical data for 77 patients in the non-progressors and progressors groups. For individual parameters of Oxford MEST classification, we found an association of score E with serum levels of IgA and Gd-IgA1 (U/mL; without neuraminidase) and with eGFR (see Median test in **S5 Table**). Composite score (M+E+S+T) was associated with serum levels of IgA and Gd-IgA1 (expressed as a relative degree of galactose deficiency measured on native IgA1, *i*.*e*., without neuraminidase) and eGFR (see **S6 Table**). ROC curve (AUC 0.936) with eGFR, biochemical markers (IgA, Gd-IgA1), and histology (individual MEST scores) shown in **S3 Fig (ROC curve a)** indicated that eGFR, serum Gd-IgA1, and histological findings (MEST scores) discriminated non-progressor and progressor groups (P values for selected variables: eGFR, 0.028; serum levels of IgA, 0.014; serum levels [U/mL] of Gd-IgA1 as a relative degree of galactose deficiency measured for IgA1 without pre-treatment with neuraminidase, 0.004; and serum levels [U/mL] of Gd-IgA1 treated with neuraminidase, 0.047). ROC curve with clinical parameters (eGFR), and histological findings (MEST scores) had an AUC of only 0.836 (**S3 Fig, ROC curve b)**.

We next developed an ordinal logit model using XLSTAT software for assessment of 77 IgAN patients with eGFR values ≥60 ml/min/1.73 m^2^ (group 1, n = 35) and those with eGFR values <60 ml/min/1.73 m^2^ (group 2, n = 42) at the time of renal biopsy and maintained during follow up (**Table 2)**. **S4 Fig** shows a ROC curve and a regression model for a limited number of explanatory variables that were significant according to discrimination analysis. When this regression model was applied to the data of the 77-patient cohort, the clasification was correct in 100%.

**Table 2:** Renal function and Oxford histological evaluation for two groups of patients with IgAN with eGFR (MDRD) values ≥60 ml/min/1.73 m^2^ (group 1) and <60 ml/min/1.73 m^2^ (group 2) at the time of renal biopsy.

**Group**	**Patients (n)**	**eGFR**	**M0**	**M1**	**E0**	**E1**	**S0**	**S1**	**T0**	**T1**	**T2**
**1**	35	97(23)	23	77	51	49	11	89	83	14	3
**2**	42	33(14)	10	90	38	62	0	100	24	55	21

eGFR values shown as mean (SD). Groups 1 and 2 denote two groups of patients with IgAN with eGFR (MDRD) values ≥60 ml/min/1.73 m^2^ and <60 ml/min/1.73 m^2^, respectively, at time of renal biopsy and maintained during follow up. Group 1 with mean eGFR 97 ml/min/1.73 m^2^, Group 2 with mean eGFR 33 ml/min/1.73 m^2^. Oxford histological scores for each group are shown; data (M0/1, E0/1, S0/1, T0/1/2) are presented as percentages of IgAN patients in each group. Patients (n), number of patients; eGFR, estimated glomerular filtration rate calculated by MDRD at renal biopsy (mean, ml/min/1.73 m^2^); SD, standard deviation.

Next, we tested whether certain levels of serum IgA and Gd-IgA1 are characteristic for 35 IgAN patients with high eGFR (group 1; patients with eGFR ≥60 mL/min/1.73 m^2^) that is maintained during the follow-up, *i*.*e*., patients who likely have a mild disease with low risk of progression, as compared to 42 IgAN patients with eGFR <60 mL/min/1.73 m^2^ at biopsy and maintained during follow up (group 2). Surprisingly, none of these biomarkers fulfilled this criterion. Therefore, we extended the analysis and measured levels of serum IgG autoantibodies specific for Gd-IgA1. This biomarker differentiated these two groups of patients (**Table 3, S4 Fig, S5 Fig**). Specifically, serum levels of IgG autoantibodies were lower in patients with eGFR ≥60 mL/min/1.73 m^2^ (group 1) than in patients eGFR <60 mL/min/1.73 m^2^ (group 2) (P = 0.003; **Table 4**). Thus, low serum levels of IgG autoantibodies were characteristic for IgAN patients with eGFR ≥60 mL/min/1.73 m^2^ at diagnosis who maintained good renal function during the follow-up. Both groups exhibited similar proteinuria at diagnosis (2.1 and 2.3 g/24 h, respectively), but patients in group 1 had lower proteinuria at the end of follow-up compared to patients in group 2 (0.7 *vs*. 1.7 g/24 h, respectively; **Table 4**). To assess the impact of treatment, we compared data for the patients treated with standard corticosteroid therapy. Notably, corticosteroid-treated patients in group 1 (n = 16) had lower proteinuria at the end of the follow-up than those in group 2 (n = 24) (0.62 vs 2.13 g/24 h, respectively, from the initial values of 1.97 *vs*. 2.67 g/24 h, respectively; **S7 Table**).

**Table 3:** Significance testing (Mann-Whitney test) of various parameters in patients with IgAN with eGFR (MDRD) ≥60 ml/min/1.73 m^2^ (group 1) or <60 ml/min/1.73 m^2^ (group 2).

Variable	p value
**S creat**	**< 0.0001**
**eGFR**	**< 0.0001**
**PU**	0.176
**IgA (μg/mL)**	0.190
**Gd-IgA1 (U/1** μg IgA)*	**0.101**
Gd-IgA1 (U/mL)*	0.117
**Gd-IgA1 (U/1** μ**g IgA)** **	0.686
Gd-IgA1 (U/mL)**	0.545
**IgG autoantibody specific for Gd-IgA1 (U/mL)**	**0.003**
**S creat**_**f**_	**< 0.0001**
**eGFR**_**f**_	**< 0.0001**
**PU**_**f**_	**< 0.0001**

* serum Gd-IgA1 without neuraminidase pretreatment

** serum Gd-IgA1 with neuraminidase pretreatment

Group 1 (n = 35), eGFR ≥60 mL/min/1.73 m^2^; group 2 (n = 42), eGFR <60 mL/min/1.73 m^2^; S creat_f_, eGFR_f_, PU_f_−final values at the end of the period of investigation (3.5 yr); S-creat–serum creatinine (μmol/L); eGFR (MDRD, mL/min/1.73 m^2^); PU- proteinuria (g/24 h). Bold numbers indicate statistically significant P values (cut-off 0.1).

**Table 4:** Average values for various parameters in the group with high eGFR that is maintained during the follow-up (group 1: 35 patients with eGFR ≥60 mL/min/1.73 m^2^) compared to group 2 (42 patients with eGFR <60 mL/min/1.73 m^2^).

Variable	Group 1	Group 2	p value***
**S creat**	83	212	**<0.0001**
**S creat**_**f1**_	88	237	**<0.0001**
**eGFR**	96	33	**<0.0001**
**eGFR**_**f1**_	85	34	**<0.0001**
Δ**eGFR**	-13%	-3%	
**PU**	2,09	2,29	0.486
**PU**_**f1**_	0,73	1,69	**0.026**
Δ**PU**	-65%	-26%	
**IgA (μg/mL)**	4 756	5 226	0.190
**Gd-IgA1 (U/1** μg IgA)*	100	115	**0.101**
Gd-IgA1 (U/mL)*	468 804	567 790	0.117
**Gd-IgA1 (U/1** μ**g IgA)** **	466	477	0.686
Gd-IgA1 (U/mL)**	2 210 341	2 371 748	0.545
**IgG Ab**	2 694	4 396	**0.003**
**S creat**_**f2**_	91		
**eGFR**_**f2**_	77		
**PU**_**f2**_	0.6		

S creat_f1_, eGFR_f1_, PU_f1_ –final values at the end of the follow-up (average 3.5 yrs); S creat_f2_, eGFR_f2_, PU_f2_ –final values at the end of the follow-up (average 5.0 yrs); S-creat, serum creatinine (μmol/L); eGFR (MDRD, mL/min/1.73 m^2^); PU, proteinuria (g/24 h); IgG Ab, serum IgG autoantibody specific for Gd-IgA1 (U/mL). Both groups exhibited similar proteinuria at diagnosis (2.1 and 2.3 g/24 h, respectively), but patients in group 1 had lower proteinuria at the end of follow-up compared to patients in group 2 (0.7 *vs*. 1.7 g/24 h, respectively). Group 1 (n = 35), eGFR ≥60 mL/min/1.73 m^2^; group 2 (n = 42), eGFR <60 mL/min/1.73 m^2^.

* serum Gd-IgA1 without neuraminidase pretreatment

** serum Gd-IgA1 with neuraminidase pretreatment

***p values means the risk to reject the null hypothesis (the medians are all equal). Confidence level is 90% (cut-off 0.1). Bold numbers indicate statistically significant P values.

As previously published [21], IgAN patients with a high risk of progression to dialysis/death had high serum levels of Gd-IgA1 at the time of diagnosis. Another study confirmed elevated serum levels of Gd-IgA1 in Japanese patients with IgAN, but association with disease progression was not assessed [22]. In the study of 62 Caucasian patients with IgAN [23], a higher serum level of Gd-IgA1 correlated with worse proteinuria; our results have not replicated this observation. In our study, we confirmed the association of Gd-IgA1-specific IgG autoantibodies in patients with IgAN with disease progression, in agreement with previous study that reported that the serum levels of IgG or IgA autoantibodies in IgAN patients associated with the absolute renal risk for ultimate dialysis or death [24]. A limitation of our study includes the calculation of glomerular filtration rate estimated with the MDRD formula, that has not been validated for eGFR ≥60 mL/min/1.73 m^2^. However, eGFR assessed with CKD-EPI formula showed similar results and no significant difference was found by Student’s t-test between eGFR estimated with the MDRD formula and with CKD-EPI formula.

In summary, our results confirmed the levels of Gd-IgA1 as an important predictor of disease progression in Czech patients with IgAN. Levels of Gd-IgA1 with and without neuraminidase pre-treatment were associated with the rate of eGFR decline. This is, to our knowledge, the first report on a higher relative degree of galactose deficiency of *native* serum IgA1 predicting a faster eGFR decline and poor renal survival. Moreover, we found that renal function (eGFR) and one biomarker (serum levels of IgG autoantibodies specific for the Gd-IgA1) at the time of diagnosis can together predict risk of disease progression. Furthermore, low serum levels of IgG autoantibodies specific for Gd-IgA1 identified patients with IgAN who maintained high eGFR and, thus, had low risk of disease progression. However, patients with high serum levels of IgG autoantibodies specific for Gd-IgA1 and other risk factors, such as high proteinuria or active histological leasions, should be indicated for immunosuppressive regimen due to suspected worse renal outcome.

## Supporting information

S1 Table Baseline characteristics of the 91 Czech patients with biopsy-proven IgA nephropathy. (DOCX)

S2 Table Baseline characteristics of the three groups of the 91 Czech patients with biopsy-proven IgA nephropathy (non-progressors, progressors, patients with ESRD). (DOCX)

S3 Table Analysis of a combined group of IgAN non-progressors and progressors *vs*. IgAN patients who reached ESRD. (DOCX)

S4 Table Median test (Mood test) comparing IgAN patients with end-stage renal disease reached during the follow-up *vs*. IgAN patients without ESRD for selected variables. (DOCX)

S5 Table Assessment of two groups (non-progressors [n = 70] and progressors [n = 7]) for the influence of E from the Oxford classification (MEST); other parameters (M, S, T) did not reach significance). (DOCX)

S6 Table Influence of Oxford classification MEST composite score in assessment of two groups (non-progressors [n = 70] and progressors [n = 7]). (DOCX)

S7 Table Mean values of selected laboratory parameters in a subset of patients treated with corticosteroids in group 1 with eGFR ≥60 mL/min/1.73 m^2^ (n = 16) and group 2 with eGFR <60 mL/min/1.73 m^2^ (n = 24). (DOCX)

S1 Fig Discriminant analysis for three groups of IgAN patients (non-progressors in green, progressors in orange, ESRD in blue) and all parameters (eGFR, using MDRD formula (mL/min/1.73 m^2^); serum IgA (mg/mL); serum Gd-IgA1 (U/1 ∝g IgA; without neuraminidase); serum Gd-IgA1 (U/mL; without neuraminidase); serum Gd-IgA1 (U/1 ∝g IgA; with neuraminidase); serum Gd-IgA1 (U/mL; with neuraminidase). (DOCX)

S2 Fig Box-and-whiskers plots for progressors and non-progressors (group 1) *vs*. IgAN patients who reached ESRD during follow up (group 2) for selected parameters. (DOCX)

S3 Fig Receiver operating characteristic (ROC) curves for non-progressors *vs.* progressors. a- ROC curve for non-progressors *vs.* progressors using eGFR (MDRD, mL/min/1.73 m^2^), Gd-IgA1 biomarkers, and Oxford classification (individual parameters of Oxford MEST classification). Area under the curve, AUC = 0.936. b- Receiver operating characteristic (ROC) curve for non-progressors *vs.* progressors using eGFR (MDRD, mL/min/1.73 m^2^) and Oxford classification (individual parameters of Oxford MEST classification). Area under the curve, AUC = 0.836.
(DOCX)

S4 Fig Receiver operator curve within two groups (eGFR, serum levels of IgG autoantibody specific for Gd-IgA1). Area under the curve = 1.00.
Accuracy of the discrimination is 100%. Group 1 (n = 35), eGFR ≥60 mL/min/1.73 m^2^ at the time of renal-biopsy; group 2 (n = 42), eGFR <60 mL/min/1.73 m^2^ at the time of renal-biopsy.
Prediction equation from logistic regression (predicts probability to choose group 1): Pred(group 1) = 1 / (1 + exp(-(1517.5–1.2E-02*AB-IgA-24.9*eGFR))).
(DOCX)

S5 Fig Box-and-whiskers plots for selected variables within two groups. S-creat, serum creatinine (μmol/L); eGFR (MDRD, mL/min/1.73 m^2^); serum IgG autoantibody specific for Gd-IgA1 (U/mL). Group 1 (n = 35), eGFR ≥60 mL/min/1.73 m^2^ at the time of renal biopsy; group 2 (n = 42), eGFR <60 mL/min/1.73 m^2^ at the time of renal biopsy.
(DOCX)
