# Correction: Galactose-deficient IgA1 and the corresponding IgG autoantibodies predict IgA nephropathy progression

**DOI:** 10.1371/journal.pone.0219947

**Published:** 2019-07-12

**Authors:** Dita Maixnerova, Chunyan Ling, Stacy Hall, Colin Reily, Rhubell Brown, Michaela Neprasova, Miloslav Suchanek, Eva Honsova, Tomas Zima, Jan Novak, Vladimir Tesar

The Supporting Information files were published with tracked changes. Please view the correct files below.

## Supporting information

S1 Table Baseline characteristics of the 91 Czech patients with biopsy-proven IgA nephropathy. (DOCX)

S2 Table Baseline characteristics of the three groups of the 91 Czech patients with biopsy-proven IgA nephropathy (non-progressors, progressors, patients with ESRD). (DOCX)

S3 Table Analysis of a combined group of IgAN non-progressors and progressors vs. IgAN patients who reached ESRD. (DOCX)

S4 Table Median test (Mood test) comparing IgAN patients with end-stage renal disease reached during the follow-up *vs*. IgAN patients without ESRD for selected variables. (DOCX)

S5 Table Assessment of two groups (non-progressors [n = 70] and progressors [n = 7]) for the influence of E from the Oxford classification (MEST); other parameters (M, S, T) did not reach significance). (DOCX)

S6 Table Influence of Oxford classification MEST composite score in assessment of two groups (non-progressors [n = 70] and progressors [n = 7]). (DOCX)

S7 Table Mean values of selected laboratory parameters in a subset of patients treated with corticosteroids in group 1 with eGFR ≥60 mL/min/1.73 m2 (n = 16) and group 2 with eGFR <60 mL/min/1.73 m2 (n = 24). (DOCX)

S1 Fig Discriminant analysis for three groups of IgAN patients (non-progressors in green, progressors in orange, ESRD in blue) and all parameters (eGFR, using MDRD formula (mL/min/1.73 m2); serum IgA (mg/mL); serum Gd-IgA1 (U/1 ∝g IgA; without neuraminidase); serum Gd-IgA1 (U/mL; without neuraminidase); serum Gd-IgA1 (U/1 ∝g IgA; with neuraminidase); serum Gd-IgA1 (U/mL; with neuraminidase). (DOCX)

S2 Fig Box-and-whiskers plots for progressors and non-progressors (group 1) *vs*. IgAN patients who reached ESRD during follow up (group 2) for selected parameters. (DOCX)

S3 Fig Receiver operating characteristic (ROC) curves for non-progressors *vs*. progressors. a- ROC curve for non-progressors *vs*. progressors using eGFR (MDRD, mL/min/1.73 m^2^), Gd-IgA1 biomarkers, and Oxford classification (individual parameters of Oxford MEST classification). Area under the curve, AUC = 0.936. b- Receiver operating characteristic (ROC) curve for non-progressors *vs*. progressors using eGFR (MDRD, mL/min/1.73 m^2^) and Oxford classification (individual parameters of Oxford MEST classification). Area under the curve, AUC = 0.836.
(DOCX)

S4 Fig Receiver operator curve within two groups (eGFR, serum levels of IgG autoantibody specific for Gd-IgA1). Area under the curve = 1.00. Accuracy of the discrimination is 100%. Group 1 (n = 35), eGFR ≥60 mL/min/1.73 m^2^ at the time of renal-biopsy; group 2 (n = 42), eGFR <60 mL/min/1.73 m^2^ at the time of renal-biopsy. Prediction equation from logistic regression (predicts probability to choose group 1): Pred(group 1) = 1 / (1 + exp(-(1517.5–1.2E-02*AB-IgA-24.9*eGFR))).
(DOCX)

S5 Fig Box-and-whiskers plots for selected variables within two groups. S-creat, serum creatinine (μmol/L); eGFR (MDRD, mL/min/1.73 m2); serum IgG autoantibody specific for Gd-IgA1 (U/mL). Group 1 (n = 35), eGFR ≥60 mL/min/1.73 m2 at the time of renal biopsy; group 2 (n = 42), eGFR <60 mL/min/1.73 m2 at the time of renal biopsy.
(DOCX)
