# Supplementary material for: Galactose-deficient IgA1 and the corresponding IgG autoantibodies predict IgA nephropathy progression
Source: PLoS One. 2019 Feb 22;14(2):e0212254. doi: 10.1371/journal.pone.0212254 (PMC6386256; doi:10.1371/journal.pone.0212254)
Supplement: S1 Table — (DOCX) [file pone.0212254.s001.docx]

**Supplemental Table 1.** Baseline characteristics of the 91 Czech patients with biopsy-proven IgA nephropathy.

| Variables – at diagnosis | Values (SD) |
| --- | --- |
| Serum creatinine (µmol/L) | 205 (182) |
| eGFR (MDRD, mL/min/1.73 m^2^) | 56 (39) |
| Proteinuria (g/24 h) | 2.3 (2.2) |
| Serum IgA (µg/mL) | 5 062 (2 305) |
| Serum Gd-IgA1 (U/1 µg IgA) without neuraminidase | 116 (63) |
| Serum Gd-IgA1 (U/1 µg IgA) with neuramidinase | 470 (275) |
| Serum Gd-IgA1 (U/mL)  without neuraminidase | 568 810 (353 501) |
| Serum Gd-IgA1 (U/mL)  with neuraminidase | 2 337 184 (1 465 651) |
| Sex [M (%)] | 74 |
| Age (years) | 43.6 (14.5) |
| Median follow-up (years) | 3.5 ± 1.1 |

Values are shown as means (SD). SD, standard deviation.
