# Supplementary material for: Galactose-deficient IgA1 and the corresponding IgG autoantibodies predict IgA nephropathy progression
Source: PLoS One. 2019 Feb 22;14(2):e0212254. doi: 10.1371/journal.pone.0212254 (PMC6386256; doi:10.1371/journal.pone.0212254)
Supplement: S2 Table — (DOCX) [file pone.0212254.s002.docx]

**Supplemental Table 2.** Baseline characteristics of the three groups of the 91 Czech patients with biopsy-proven IgA nephropathy (non-progressors, progressors, patients with ESRD)

**Variables – at diagnosis Values (SD)**

NONPROG PROG ESRD

____________________________________________________________________________________________

**S-creat** 142 (80) 211 (87) 517 (254)

**S-creat_f_** 149 (93) 438 (129) 847 (209)

eGFR 65 (37) 34 (13) 24 (34)

eGFR_f_ 59 (34) 13 (4.8) 7.0 (4.1)

PU 2.3 (2.2) 1.7 (1.6) 2.7 (2.7)

Pu_f_ 1.3 (1.6) 2.3 (1.3) non-measured

[IgA] (ug/mL) 4811 (2011) 6862 (2551) 5420 (3179)

Gd-IgA1 (U/1 ug IgA; without neuraminidase) 109 (63) 139 (66) 139 (59)

Gd-IgA1 (U/mL; without neuraminidase) 504783 (297010) 846111 (251279) 750295 (518475)

Gd-IgA1 (U/1 ug IgA; with neuraminidase) 471 (282) 587 (359) 405 (179)

Gd-IgA1 (U/mL; with neuraminidase) 2260775 (1469390) 3415807 (1114505) 2179918 (1467646)

IgG auto-Ab (U/mL) 3592 (2190) 2960 (2047) 4144 (2821)

NONPROG - non-progressors (n=70, with stable renal function during follow-up), PROG - progressors (n=7, defined by the decline of eGFR ≥50% from baseline, but with eGFR >15ml/min/1.73 m^2^ at the end of follow-up), ESRD - patients who reached end-stage renal disease during follow-up (n=14, defined as those with serum creatinine >400 µmol/L at diagnosis, eGFR <15 ml/min/1.73 m^2^ at the end of follow-up, or those who started renal replacement therapy).

Values are shown as means (SD). SD, standard deviation.
