# Supplementary material for: Galactose-deficient IgA1 and the corresponding IgG autoantibodies predict IgA nephropathy progression
Source: PLoS One. 2019 Feb 22;14(2):e0212254. doi: 10.1371/journal.pone.0212254 (PMC6386256; doi:10.1371/journal.pone.0212254)
Supplement: S3 Table — (DOCX) [file pone.0212254.s003.docx]

**Supplemental Table 3.** Analysis of a combined group of IgAN non-progressors and progressors *vs.* IgAN patients who reached ESRD.

| Variables – at diagnosis | Non-progressors+ progressors | ESRD |
| --- | --- | --- |
| Percentage of all subjects (%) | 85 | 15 |
| Serum creatinine (µmol/L) | 149 (83) | 517 (254) |
| eGFR (MDRD, mL/min/1.73 m^2^) | 62 (37) | 24 (34) |
| Proteinuria (g/24 h) | 2.2 (2.1) | 2.7 (2.7) |
| Serum IgA (µg/mL) | 4 997 (2 130) | 5 420 (3 179) |
| Serum Gd-IgA1 (U/1 µg IgA)  without neuraminidase | 111 (63) | 139 (59) |
| Serum Gd-IgA1 (U/1 µg IgA)  with neuraminidase | 482 (289) | 405 (179) |
| Serum Gd-IgA1 (U/mL)  without neuraminidase | 535 813 (307 945) | 750 295 (518 475) |
| Serum Gd-IgA1 (U/mL)  with neuraminidase | 2 365 778 (1 473 096) | 2 179 918 (1 467 646) |

Values are shown as means and SD, standard deviation; non-progressors (patients with stable renal parameters); progressors (patients with decline of eGFR ≥50 % above baseline; ESRD (patients with end-stage renal disease reached during the follow-up, patients with eGFR<15 ml/min/1.73 m^2^ at the end of follow-up or patients who started renal replacement therapy at the end of follow-up).
