# Supplementary material for: Galactose-deficient IgA1 and the corresponding IgG autoantibodies predict IgA nephropathy progression
Source: PLoS One. 2019 Feb 22;14(2):e0212254. doi: 10.1371/journal.pone.0212254 (PMC6386256; doi:10.1371/journal.pone.0212254)
Supplement: S4 Table — (DOCX) [file pone.0212254.s004.docx]

**Supplemental Table 4.** Median test (Mood test) comparing IgAN patients with end-stage renal disease reached during the follow-up *vs.* IgAN patients without ESRD for selected variables.

| Variable | p value*** |
| --- | --- |
| **S creat** | **0.0003** |
| **eGFR** | **0.003** |
| **IgA (µg/mL)** | 0.195 |
| **Gd-IgA1 (U/1 µg IgA)*** | **0.049** |
| **Gd-IgA1 (U/mL)*** | 0.120 |
| **Gd-IgA1 (U/1 µg IgA) **** | 0.227 |
| **Gd-IgA1 (U/mL)**** | 0.227 |

* serum Gd-IgA1 without neuraminidase pretreatment

** serum Gd-IgA1 with neuraminidase pretreatment

***p values mean the risk to reject the null hypothesis (the medians are all equal). Confidence level is 90% (cut-off 0.1). Bold numbers indicate statistically significant P values.

S-creat, serum creatinine (µmol/L); eGFR (MDRD, mL/min/1.73 m^2^)
