# Supplementary material for: Galactose-deficient IgA1 and the corresponding IgG autoantibodies predict IgA nephropathy progression
Source: PLoS One. 2019 Feb 22;14(2):e0212254. doi: 10.1371/journal.pone.0212254 (PMC6386256; doi:10.1371/journal.pone.0212254)
Supplement: S6 Table — (DOCX) [file pone.0212254.s006.docx]

**Supplemental Table 6.** Influence of Oxford classification MEST composite score in assessment of two groups (non-progressors [n=70] and progressors [n=7])

| Variable | p value*** |
| --- | --- |
| **S creat** | **<0.0001** |
| **eGFR** | **0.0001** |
| **IgA (µg/mL)** | **0.024** |
| **Gd-IgA1 (U/1 µg IgA)*** | **0.101** |
| **Gd-IgA1 (U/mL)*** | 0.513 |
| **Gd-IgA1 (U/1 µg IgA)**** | 0.534 |
| **Gd-IgA1 (U/mL)**** | 0.147 |

* serum Gd-IgA1 without neuraminidase pretreatment

** serum Gd-IgA1 with neuraminidase pretreatment

***p values means the risk to reject the null hypothesis (the medians are all equal). Confidence level is 90% (cut-off 0.1). Bold numbers indicate statistically significant P values.

S-creat, serum creatinine (µmol/L); eGFR (MDRD, mL/min/1.73 m^2^)
