# Supplementary material for: Galactose-deficient IgA1 and the corresponding IgG autoantibodies predict IgA nephropathy progression
Source: PLoS One. 2019 Feb 22;14(2):e0212254. doi: 10.1371/journal.pone.0212254 (PMC6386256; doi:10.1371/journal.pone.0212254)
Supplement: S7 Table — (DOCX) [file pone.0212254.s007.docx]

**Supplemental Table 7.** Mean values of selected laboratory parameters in a subset of patients treated with corticosteroids in group 1 with eGFR ≥60 mL/min/1.73 m^2^ (n = 16) and group 2 with eGFR <60 mL/min/1.73 m^2^ (n = 24).

|  | S creat | eGFR | PU | S creat_f_ | eGFR_f_ | PU_f_ |
| --- | --- | --- | --- | --- | --- | --- |
| Group 1 eGFR >60 (n=16) | 75 | 108 | 1.97 | 84 | 87 | 0.62 |
| Group 2 eGFR <60 (n=24) | 234 | 29 | 2.67 | 241 | 33 | 2.13 |

S creat_f_, eGFR_f_, PU_f_, final values at the end of the period of follow-up (for group 1, 5.0 yrs.; for group 2, 3.5 yrs.)

S-creat, serum creatinine (µmol/L); eGFR (MDRD, mL/min/1.73 m^2^); PU, proteinuria (g/24 h)

To assess the impact of treatment, we compared data for the patients treated with standard corticosteroid therapy. Notably, corticosteroid-treated patients in group 1 (n=16) had lower proteinuria at the end of the follow-up than those in group 2 (n=24) (0.62 *vs.* 2.13 g/24 h, respectively, from the initial values of 1.97 *vs.* 2.67 g/24 h, respectively.
