# Supplementary material for: Galactose-deficient IgA1 and the corresponding IgG autoantibodies predict IgA nephropathy progression
Source: PLoS One. 2019 Feb 22;14(2):e0212254. doi: 10.1371/journal.pone.0212254 (PMC6386256; doi:10.1371/journal.pone.0212254)
Supplement: S1 Fig — (DOCX) [file pone.0212254.s008.docx]

**Supplemental Figure 1.** Discriminant analysis for three groups of IgAN patients (non-progressors in green, progressors in orange, ESRD in blue) and all parameters (eGFR, using MDRD formula (mL/min/1.73 m^2^); serum IgA (mg/mL); serum Gd-IgA1 (U/1 μg IgA; without neuraminidase); serum Gd-IgA1 (U/mL; without neuraminidase); serum Gd-IgA1 (U/1 μg IgA; with neuraminidase); serum Gd-IgA1 (U/mL; with neuraminidase). Non-progressors (patients with stable renal parameters), progressors (patients with decline of eGFR ≥50% above baseline, ESRD (patients with end-stage renal disease reached during the follow-up, patients with eGFR<15 ml/min/1.73 m^2^ at the end of follow-up or patients who started renal replacement therapy at the end of follow-up).
