# Supplementary material for: Galactose-deficient IgA1 and the corresponding IgG autoantibodies predict IgA nephropathy progression
Source: PLoS One. 2019 Feb 22;14(2):e0212254. doi: 10.1371/journal.pone.0212254 (PMC6386256; doi:10.1371/journal.pone.0212254)
Supplement: S2 Fig — (DOCX) [file pone.0212254.s009.docx]

S-creatinine

µmol/L

eGFR (MDRD)

mL/min/1.73 m^2^

2

1

Gd-IgA1 (without neuraminidase)

U/1 µg IgA

**Supplemental Figure 2.** Box-and-whiskers plots for progressors and non-progressors (group 1) *vs.* IgAN patients who reached ESRD during follow up (group 2) for selected parameters.
