# Supplementary material for: Galactose-deficient IgA1 and the corresponding IgG autoantibodies predict IgA nephropathy progression
Source: PLoS One. 2019 Feb 22;14(2):e0212254. doi: 10.1371/journal.pone.0212254 (PMC6386256; doi:10.1371/journal.pone.0212254)
Supplement: S3 Fig — Receiver operating characteristic (ROC) curves for non-progressors vs. progressors. a- ROC curve for non-progressors vs. progressors using eGFR (MDRD, mL/min/1.73 m2), Gd-IgA1 biomarkers, and Oxford classification (individual parameters of Oxford MEST classification). Area under the curve, AUC = 0.936. b- Receiver operating characteristic (ROC) curve for non-progressors vs. progressors using eGFR (MDRD, mL/min/1.73 m2) and Oxford classification (individual parameters of Oxford MEST classification). Area under the curve, AUC = 0.836. (DOCX) [file pone.0212254.s010.docx]

**bab**

**a**

**Supplemental Figure 3.** Receiver operating characteristic (ROC) curves for non-progressors *vs.* progressors. **a-** ROC curve for non-progressors *vs.* progressors using eGFR (MDRD, mL/min/1.73 m^2^), Gd-IgA1 biomarkers, and Oxford classification (individual parameters of Oxford MEST classification). Area under the curve, AUC = 0.936. **b-** Receiver operating characteristic (ROC) curve for non-progressors *vs.* progressors using eGFR (MDRD, mL/min/1.73 m^2^) and Oxford classification (individual parameters of Oxford MEST classification). Area under the curve, AUC = 0.836.

eGFR (MDRD, mL/min/1.73 m^2^); IgA and Gd-IgA1 biomarkers: serum IgA (µg/mL); serum Gd-IgA1 (U/1 µg IgA) without neuraminidase; serum Gd-IgA1 (U/1 µg IgA) with neuraminidase; serum Gd-IgA1 (U/mL) without neuraminidase; serum Gd-IgA1 (U/mL) with neuraminidase.
