# Supplementary material for: Galactose-deficient IgA1 and the corresponding IgG autoantibodies predict IgA nephropathy progression
Source: PLoS One. 2019 Feb 22;14(2):e0212254. doi: 10.1371/journal.pone.0212254 (PMC6386256; doi:10.1371/journal.pone.0212254)
Supplement: S4 Fig — Area under the curve = 1.00. Accuracy of the discrimination is 100%. Group 1 (n = 35), eGFR ≥60 mL/min/1.73 m2 at the time of renal-biopsy; group 2 (n = 42), eGFR <60 mL/min/1.73 m2 at the time of renal-biopsy. Prediction equation from logistic regression (predicts probability to choose group 1): Pred(group 1) = 1 / (1 + exp(-(1517.5–1.2E-02*AB-IgA-24.9*eGFR))). (DOCX) [file pone.0212254.s011.docx]

**Supplemental Figure 4.** Receiver operator curve within two groups (eGFR, serum levels of IgG autoantibody specific for Gd-IgA1). Area under the curve = 1.00. Accuracy of the discrimination is 100%.

Group 1 (n = 35), eGFR >60 mL/min/1.73 m^2^ at the time of renal-biopsy; group 2 (n = 42), eGFR <60 mL/min/1.73 m^2^ at the time of renal-biopsy.

Prediction equation from logistic regression (predicts probability to choose group 1):

Pred(group 1) = 1 / (1 + exp(-(1517.5-1.2E-02****AB-IgA***-24.9****eGFR***))).
