# Supplementary material for: Galactose-deficient IgA1 and the corresponding IgG autoantibodies predict IgA nephropathy progression
Source: PLoS One. 2019 Feb 22;14(2):e0212254. doi: 10.1371/journal.pone.0212254 (PMC6386256; doi:10.1371/journal.pone.0212254)
Supplement: S5 Fig — S-creat, serum creatinine (μmol/L); eGFR (MDRD, mL/min/1.73 m2); serum IgG autoantibody specific for Gd-IgA1 (U/mL). Group 1 (n = 35), eGFR ≥60 mL/min/1.73 m2 at the time of renal biopsy; group 2 (n = 42), eGFR <60 mL/min/1.73 m2 at the time of renal biopsy. (DOCX) [file pone.0212254.s012.docx]

S creat

Gd-IgA1-specific IgG autoantibodies

eGFR

**Supplemental Figure 5.** Box-and-whiskers plots for selected variables within two groups.

S-creat, serum creatinine (µmol/L); eGFR (MDRD, mL/min/1.73 m^2^); serum IgG autoantibody specific for Gd-IgA1 (U/mL). Group 1 (n = 35), eGFR >60 mL/min/1.73 m^2^ at the time of renal biopsy; group 2 (n = 42), eGFR <60 mL/min/1.73 m^2^ at the time of renal biopsy.
